# Emergency departments in the United States treating high proportions of patients with ambulatory care sensitive conditions: a retrospective cross-sectional analysis

**DOI:** 10.1186/s12913-022-08240-7

**Published:** 2022-07-02

**Authors:** Charleen Hsuan, Alexis Zebrowski, Michelle P. Lin, David G. Buckler, Brendan G. Carr

**Affiliations:** 1 Department of Health Policy and Administration, Pennsylvania State University, 601B Ford Building, University Park, PA 16802 USA; 2 Department of Emergency Medicine, Icahn School of Medicine at Mount Sinai, New York, NY USA

**Keywords:** Ambulatory care sensitive conditions, Safety net hospitals, Emergency departments

## Abstract

**Background:**

One in nine emergency department (ED) visits by Medicare beneficiaries are for ambulatory care sensitive conditions (ACSCs). This study aimed to examine the association between ACSC ED visits to hospitals with the highest proportion of ACSC visits (“high ACSC hospitals) and safety-net status.

**Methods:**

This was a cross-sectional study of ED visits by Medicare fee-for-service beneficiaries ≥ 65 years using 2013–14 claims data, Area Health Resources File data, and County Health Rankings. Logistic regression estimated the association between an ACSC ED visit to high ACSC hospitals, accounting for individual, hospital, and community factors, including whether the visit was to a safety-net hospital. Safety net status was measured by Disproportionate Share Hospital (DSH) index patient percentage; public hospital status; and proportion of dual-eligible beneficiaries. Hospital-level correlation was calculated between ACSC visits, DSH index, and dual-eligible patients. We stratified by type of ACSC visit: acute or chronic.

**Results:**

Among 5,192,729 ACSC ED visits, the odds of visiting a high ACSC hospital were higher for patients who were Black (1.37), dual-eligible (1.18), and with the highest comorbidity burden (1.26, *p* < 0.001 for all). ACSC visits had increased odds of being to high ACSC hospitals if the hospitals were high DSH (1.43), served the highest proportion of dual-eligible beneficiaries (2.23), and were for-profit (relative to non-profit) (1.38), and lower odds were associated with public hospitals (0.64) (*p* < 0.001 for all). This relationship was similar for visits to high chronic ACSC hospitals (high DSH: 1.59, high dual-eligibility: 2.60, for-profit: 1.41, public: 0.63, all *p* < 0.001) and to a lesser extent, high acute ACSC hospitals (high DSH: 1.02; high dual-eligibility: 1.48, for-profit: 1.17, public: 0.94, *p* < 0.001). The proportion of ACSC visits at all hospitals was weakly correlated with DSH proportion (0.2) and the proportion of dual-eligible patients (0.29), and this relationship was also seen for both chronic and acute ACSC visits, though stronger for the chronic ACSC visits.

**Conclusion:**

Visits to hospitals with a high proportion of acute ACSC ED visits may be less likely to be to hospitals classified as safety net hospitals than those with a high proportion of chronic ACSC visits.

**Supplementary Information:**

The online version contains supplementary material available at 10.1186/s12913-022-08240-7.

## Background

Ambulatory care sensitive conditions (ACSCs), or conditions for which outpatient care can potentially prevent hospital visits, [1] account for 11% to 14% of emergency department (ED) visits by Medicare beneficiaries [2, 3]. In the United States, Medicare is a federal program that is the primary source of health insurance for older adults (65 and above). The U.S. Agency for Healthcare Quality and Research divides ACSCs for ages 18 and above into acute ACSCs, conditions for which clinical deterioration requiring hospital visits could have been prevented by early outpatient intervention (e.g., bacterial pneumonia), and chronic ACSCs, conditions for which timely and well-coordinated disease management could have prevented exacerbations (e.g., diabetes complication). Because of their importance in reflecting the quality of primary and preventive care, Medicare has incorporated ACSCs into its physician pay-for-performance program [4]. Although ACSCs accounted for $29.6 billion in hospital care in the United States in 2010 ($36.2 billion in 2021 dollars), [5] little research has characterized the hospitals serving a high proportion of ACSC ED visits or whether these hospitals are classified as safety-net hospitals.

Patients and communities with limited access to care are often treated at safety-net hospitals [6]. Safety-net facilities share a mission to “deliver a significant level of health care to uninsured, Medicaid, and other vulnerable patients,” [7] but may differ with respect to ownership, size, location, and scope of services provided. There is little consensus on how to measure or define hospital safety net status and low concordance between the definitions [8, 9]. Medicare reimbursement policy identifies safety-net hospitals using the Disproportionate Share Hospital (DSH) index [10]. The DSH adjustment is calculated based on inpatient care, either under the primary method based on the hospital’s disproportionate patient percentage of low-income inpatients, or under the alternative special exception method based on the percent of inpatient care revenues from state and local government sources for indigent care [11]. Other ways to characterize safety-net hospitals are ownership status (public hospitals) and the proportion of patients with dual-eligibility for Medicare and Medicaid (“dual-eligible”) treated, which is used as an indicator of social disadvantage by other Medicare policy such as the Hospital Readmissions Reduction Program. Medicaid is a joint federal and state insurance program that covers low-income individuals, so dual-eligible patients are a particularly vulnerable population that have a higher number of chronic conditions, lower health status, and on average, are disproportionately high cost [12, 13].

Our study objective was to understand the association between visiting a hospital that treats a high proportion of ACSC ED visits with visiting a safety-net hospital. Specifically, previous research suggests patients who are more vulnerable – e.g., older, minority, publicly insured, and those who live in higher poverty areas – are at higher risk for ACSC ED visits [3, 14]. Other research suggests that hospitals in communities with more minorities, lower primary care provider density, and those in rural areas have disproportionately higher rates of ACSC-related ED visits [15–17]. Common explanations for why vulnerable patients are more likely to make ACSC ED visits is lack of primary care or continuity of care; changes in primary care workforce, for instance, is associated with fewer ACSC ED visits [18]. However, little is known about ACSC visits made to hospitals with the highest proportion of ACSC ED visits. It is unknown whether visits to hospitals with a high proportion of ACSC ED visits are also associated with visits to safety-net hospitals, particularly to hospitals that are defined as a safety-net hospital by Medicare financing policy.

We examined patient, hospital and neighborhood-level factors associated with hospitals having the highest proportion of ACSC ED visits among Medicare beneficiaries, both overall and separately for chronic and acute ACSCs. We separately examined chronic and acute ACSCs because of research suggesting patients making chronic ACSC visits may differ from those making acute ACSC visits [3]. We then tested the association between hospitals with the highest proportion of ACSC ED visits and safety net status, defined three ways: by DSH status, those serving a high proportion of patients with dual-eligibility, and public hospitals. We hypothesized that there would be an association between the proportion of ACSC visits and hospital safety net status for communities with increased vulnerability, but that this association would differ depending on the specific definition used.

## Methods

### Data and sample

We utilized Medicare claims data (January 2013 through December 2014) aggregated by hospital and merged with hospital characteristics from the American Hospital Association’s Annual Survey (2013–14) and community level characteristics from the Area Health Resources File (2013–14) and County Health Rankings (2013–14) [19]. Medicare data included the Medicare beneficiary denominator file containing demographic patient information, the Medicare Analysis Provider and Review (MedPAR) and outpatient research identifiable files, and the 2013–14 CMS’ New Stratified Methodology Impact File, which we used to determine disproportionate share hospital (DSH) patient percentage.

To define the cohort, we began with ED visits (regardless of whether admitted) made by fee-for-service Medicare beneficiaries aged 65 and over that met the CMS criteria for an ACSC visit [4]. (Appendix I) We excluded hospitals with 9 or fewer ACSC ED visits (*n* = 3,678 visits and 848 hospitals) because of our interest in hospitals providing a high proportion of ACSC ED visits and federal hospitals (*n* = 14,974 visits and 13 hospitals) because these hospitals primarily serve active military or veterans. We also excluded hospitals with an unknown bed count (*n* = 36,787 visits and 97 hospitals) and hospitals with fewer than 50 beds (*n* = 223,553 visits and 553 hospitals) because the distribution of ACSC visits for hospitals with fewer than 50 beds had clear differences (e.g. longer tails) compared to hospitals with more than 50 beds. (Appendix II) Hospitals with missing data in any of the predictors of interest were dropped from those analyses, but were eligible for secondary analyses if data were available.

### Defining a high ACSC hospital

ED visits for an ACSC condition were identified using ICD-9-CM diagnosis codes, based on Medicare definitions [4]. We then stratified ACSC visits by whether they were associated with a chronic or acute ACSC [4]. The three acute ACSCs are: community-acquired bacterial pneumonia, urinary tract infections, and dehydration. The six chronic ACSCs are: short term complications from diabetes, long term complications from diabetes, uncontrolled diabetes, lower extremity amputation among patients with diabetes, chronic obstructive pulmonary disease or asthma in older adults, and congestive heart failure. We then calculated for each hospital the proportion of Medicare ED visits that were associated with any ACSC, and classified a hospital as a “high ACSC hospital” if it was in the 80^th^ percentile. The 80^th^ percentile was used to define the high ACSC hospitals because of differences in how covariates were distributed above and below that percentile. In sensitivity analyses, we tested different definitions of a high ACSC hospital, based on 75^th^ and 90^th^ percentile cut-offs. (Appendix III) We then made the same calculation for chronic and acute ACSCs, and classified hospitals as “high chronic ACSC hospitals” and “high acute ACSC hospitals.”

### Variables

Our primary outcome of interest was a dichotomous variable for whether a visit occurred at a high ACSC hospital, versus not a high ACSC hospital. (see above) The secondary outcomes of interest were whether a visit occurred at a high chronic ACSC hospital, versus not, or a high acute ACSC hospital, versus not.

The primary predictor of interest was hospital safety net status. Because of the multiple definitions for safety net status, we used three different definitions: (i) hospitals with the highest quartile of the DSH patient percentage; (ii) public hospitals; and (iii) EDs serving the highest quintile of age-eligible Medicare patients with dual-eligibility for Medicare and Medicaid [20–23].

Secondary predictors of interest were patient, hospital, and community factors. Patient-level factors were: age, sex, race/ethnicity, dual-eligibility, and comorbidities. We used the Elixhauser comorbidity index, which categorizes comorbidities based on administrative data [24]. Hospital-level factors were hospital ownership, teaching status, and US Census region. Hospitals were classified as either major teaching hospitals, if they were members of the Council of Teaching Hospitals (COTH), or minor teaching hospitals, if they were non-COTH hospitals that reported a medical school affiliation to the American Medical Association. We did not examine bed size due to similar distributions across all hospitals in our cohort.

Community factors were county-level urban–rural classification (truncated version of the National Center for Health Statistics continuum [25]), health care supply (number of federally qualified health centers per 100,000, primary care physicians per 100,000), access to care (not seeing a doctor because of cost), and area socio-demographics (percent Black, percent Hispanic, percent White, percent poverty, median household income, high housing costs (percent spending 30% or more of household income on housing), percent with a high school degree or higher).

### Statistical analyses

In unadjusted analyses, we compared patient, hospital, and community factors across high ACSC hospitals. In adjusted analyses, we estimated the odds of an ED visit being to a high ACSC hospital for patient, hospital, and community factors, using logistic regression with robust standard errors (Huber/White/sandwich estimators) to account for possible heteroscedasticity [26]. We calculated 95% confidence intervals using these robust standard errors. We then ran three separate, stratified logistic models for each measure of safety net status to test the association between ACSC visits to these hospitals after adjusting for patient, hospital, and community factors. All models controlled for the same factors, except that, given the potential for collinearity, we excluded dual-eligibility for the model using the hospital’s proportion of dually-eligible patients as a definition of a safety-net hospital. Next, we examined the hospital-level Pearson correlation between the proportion of ACSC visits to the hospital, the DSH index, and the proportion of patients who were dual-eligible (ownership was excluded for correlation measures). We interpreted a correlation of less than 0.10 as negligible, 0.10–0.39 as weak, 0.40–0.69 as moderate, 0.70–0.89 as strong, and above 0.9 as very strong [27]. Finally, we stratified all analyses by whether the ACSCs visits were to high chronic ACSC hospitals or high acute ACSC hospitals.

We followed the STROBE checklist for observational studies. All analyses were conducted using STATA 15.1 (StataCorp, College Station, TX). The study was approved by institutional review boards at Thomas Jefferson University and the Icahn School of Medicine at Mount Sinai.

#### Role of the funding source

This study was funded and supported by the Agency for Healthcare Quality and Research and the National Institutes of Health, neither of which had a role in the study’s design, conduct, or reporting.

## Results

### Descriptive (unadjusted) statistics

The final sample consisted of 5,192,729ACSC ED visits among 3,670,027 unique Medicare beneficiaries at 2,722 hospitals. The median age was 78 years (IQR 72–85) and 61.3% were female (Table 1). Black patients accounted for a larger proportion of ACSC ED visits at hospitals with the highest proportion of ACSCs (18.0%) and chronic ACSCs (21.1%) relative to the total sample (13.5%). Similarly, dual-eligible beneficiaries accounted for a larger proportion of ED visits at hospitals with the highest quintile for all ACSCs (29.7%) and chronic ACSC (30.6%) relative to the entire sample (24.3%). Major teaching hospitals accounted for a larger proportion of hospitals in the highest quintile for all (73.0%), acute (74.1%) and chronic (67.7%) ACSCs relative to the total sample (62.5%). Hospitals in the highest quintile for all ACSCs (32.4%) and chronic ACSCs (36.0%), were also more likely to be in the top quartile of DSH relative to the total sample (25.9%). Among community factors, median household income and primary care provider density were lower in zip codes for hospitals in the highest quintile for all, chronic, and acute ACSCs relative to the total sample. Because of the large sample size, all of these differences were statistically significant.

**Table 1:** Characteristics of ACSC ED visits by Medicare Patients to All Hospitals and High ACSC Hospitals

	All hospitals	EDs w/ high proportion of ACSCs	EDs w/ high proportion of acute ACSCs	EDs w/ high proportion of chronic ACSCs
**N**	2,722	559	555	553
**Unique visits (N)**	5,192,729	1,009,543	997,439	1,019,814
**Unique patients (N)**	3,670,027	710,058	719,112	712,689
**Patient characteristics**				
Median age (IQR)	78 (72–85)	78 (71–85)	79 (72–86)	78 (71–85)
Age				
65–74	1,844,151 (35.5%)	372,865 (36.9%)	349,918 (35.1%)	383,138 (37.6%)
75–84	1,874,734 (36.1%)	367,253 (36.4%)	361,901 (36.3%)	369,433 (36.2%)
85 +	1,473,844 (28.4%)	269,425 (26.7%)	285,620 (28.6%)	267,243 (26.2%)
Female	3,183,433 (61.3%)	627,141 (62.1%)	622,502 (62.4%)	627,180 (61.5%)
Race				
White	4,179,259 (80.5%)	763,046 (75.6%)	824,608 (82.7%)	737,814 (72.3%)
Black	711,134 (13.7%)	181,526 (18.0%)	113,997 (11.4%)	215,608 (21.1%)
Other	302,336 (5.8%)	64,971 (6.4%)	58,834 (5.9%)	66,392 (6.5%)
Comorbidity count (Elixhauser)	3 (2–5)	4 (2–5)	3 (2–5)	4 (2–5)
Elixhauser comorbidities				
0–1	995,837 (19.2%)	168,684 (16.7%)	189,980 (19.0%)	157,065 (15.4%)
2	796,795 (15.3%)	154,055 (15.3%)	156,843 (15.7%)	151,288 (14.8%)
3 +	3,400,097 (65.6%)	686,804 (68.0%)	650,616 (65.2%)	711,461 (69.8%)
Dual-eligible	1,261,508 (24.3%)	300,022 (29.7%)	257,399 (25.8%)	311,754 (30.6%)
**Hospital characteristics**				
Teaching Hosp				
None	785 (28.8%)	131 (24.3%)	126 (24.2%)	150 (26.2%)
Minor teaching hospital (AMA)	236 (8.7%)	15 (2.8%)	9 (1.7%)	35 (6.1%)
Major teaching hospital (COTH)	1,701 (62.5%)	394 (73.0%)	386 (74.1%)	388 (67.7%)
Bed Size				
50–99	568 (20.9%)	142 (26.3%)	152 (29.2%)	123 (21.5%)
100–199	879 (32.3%)	193 (35.7%)	202 (38.8%)	199 (34.7%)
200 +	1,275 (46.8%)	205 (38.0%)	167 (32.1%)	251 (43.8%)
Ownership				
For-profit	511 (19.7%)	137 (26.9%)	121 (24.6%)	142 (26.5%)
Public	354 (13.6%)	57 (11.2%)	62 (12.6%)	63 (11.8%)
Non-profit	1,736 (66.7%)	316 (62.0%)	308 (62.7%)	330 (61.7%)
Safety net				
Highest quartile of DSH	692 (25.9%)	175 (32.4%)	131 (25.1%)	206 (36.0%)
**Community characteristics**				
Region				
Northeast	473 (17.4%)	107 (19.8%)	88 (16.9%)	132 (23.0%)
Midwest	659 (24.2%)	114 (21.1%)	113 (21.7%)	127 (22.2%)
South	1,074 (39.5%)	280 (51.9%)	260 (49.9)	276 (48.2%)
West	516 (19.0%)	39 (7.22%)	60 (11.5%)	38 (6.6%)
Urbanicity (NCHS, 2013)				
Large central metro	649 (23.9%)	132 (24.5%)	88 (17.0%)	169 (29.6%)
Large fringe metro	587 (21.6%)	127 (23.6%)	125 (24.1%)	129 (22.6%)
Medium/Small metro	879 (32.3%)	117 (21.8%)	148 (28.5%)	130 (22.8%)
Non-metro	604 (22.2%)	162 (30.1%)	158 (30.4%)	142 (25.0%)
**Hospital community characteristics** Median (IQR)				
% Poverty	15.6 (12.0–18.7)	17.4 (13.9–20.1)	16.5 (13.1–19.1)	17.4 (13.9–20.3)
Median household income	50,774 (43,103–58,539)	46,070.5 (40,530–53,624)	47,123 (40,751–55,686)	47,024 (40,957–53,795)
Didn’t see doctor because of cost	13.8 (10.7–17.2)	14.8 (12.4–18.4)	14.5 (11.5–17.8)	14.7 (12.2–17.9)
High housing costs	34.4 (28.8–40.5)	31.7 (26.9–38.5)	30.5 (26.3–36.8)	33.8 (28.3–41.1)
High school education or higher	86.9 (82.5–89.7)	84.4 (78.7–88.4)	85.0 (79.7–88.9)	84.8 (79.6–88.2)
Food insecurity	15.3 (12.7–17.6)	16.1 (13.6–17.9)	15.3 (12.9–17.6)	16.2 (13.6–18.3)
Federally qualified health centers /100 K	1.3 (0.5–2.7)	1.4 (0.5–2.8)	1.3 (0.4–2.8)	1.4 (0.5–2.8)
Primary care / 100 K	72.1 (53.5–92.7)	61.3 (45.1–82.1)	61.1 (44.9–81.5)	65.5 (48.2–85.0)
% White	79.5 (64.2–89.4)	79.9 (63.0–91.8)	81.7 (68.9–91.8)	76.2 (62.1–89.7)
% Black	8.3 (2.7–18.6)	10.5 (2.8–22.2)	7.2 (2.5–18.7)	12.5 (4.1–24.4)
% Hispanic	7.6 (3.4–19.6)	5.5 (2.3–19.1)	5.7 (2.4–17.1)	6.7 (2.6–19.7)

*Notes*. “*ED*” emergency department, “*ACSCs*” ambulatory care sensitive conditions, “*DSH*” Disproportionate Share Hospital

All patient characteristics are at the visit level; all hospital, community, and hospital community characteristics are at the hospital level

### Adjusted analyses

Compared to ACSC ED visits by white beneficiaries, ACSC ED visits by black beneficiaries were more likely to be to high ACSC hospitals (adjusted odds ratio (aOR) = 1.37, 95% confidence interval (CI): 1.36–1.38) and high chronic ACSC hospitals (aOR: 1.61, 95% CI: 1.60–1.62), and less likely to be to high acute ACSC hospitals (aOR: 0.81, 95% CI: 0.80–0.81). (Table 2), A “dose–response” effect was seen between Elixhauser comorbid conditions and ACSC visits across all three ACSC hospital categories, with the odds greatest for those with three or more comorbidities: high overall ACSCs (aOR: 1.26, 95% CI: 1.26–1.27), chronic ACSCs (aOR: 1.39, 95% CI: 1.38–1.40), and acute ACSC hospitals (aOR: 1.04, 95% CI: 1.03–1.05). ACSC ED visits were less likely to be to high ACSC hospitals if they were in the West, South, and Midwest, relative to the Northeast, region of the country (West: aOR: 0.17, 95% CI: 0.17–0.17; South: aOR: 0.38, 95% CI: 0.38–0.39; Midwest: aOR: 0.49, 95% CI: 0.49–0.50). ACSC ED visits were also more likely to be to high ACSC hospitals if they were to large fringe metropolitan hospitals (aOR: 1.58, 95% CI: 1.57–1.59) relative to large central metropolitan hospitals, and less likely if they were to medium, small, and non-metropolitan areas. Nearly all community-level factors were also significant given the sample size, however the magnitude of association for all factors was less than 0.05 (Table 2).

**Table 2:** Patient, Hospital, and Community Factors Associated with an ACSC ED visit to High ACSC Hospitals

	High ACSC Hospital aOR [95% CI]	High Chronic ACSC Hospital aOR [95% CI]	High Acute ACSC Hospital aOR [95% CI]
**Patient Characteristics**			
Age			
65–74	Ref	Ref	Ref
75–84	0.99 [0.98, 0.99]***	0.96 [0.95, 0.96]***	1.03 [1.02, 1.03]***
85 +	0.95 [0.94, 0.96] ***	0.89 [0.89, 0.90]***	1.06 [1.05, 1.07]***
Female	1.02 [1.02, 1.03]***	0.99 [0.99, 1.00]**	1.04 [1.03, 1.04]***
Race			
White	Ref	Ref	Ref
Black	1.37 [1.36, 1.38]***	1.61 [1.60, 1.62]***	0.81 [0.80, 0.82]***
Other	1.37 [1.36, 1.38]***	1.28 [1.27, 1.30]***	1.16 [1.15, 1.17]***
Elixhauser comorbidity index			
0–1	Ref	Ref	Ref
2	1.17 [1.16, 1.17]***	1.22 [1.21, 1.24]***	1.04 [1.04, 1.05]***
3 +	1.26 [1.26, 1.27]***	1.39 [1.38, 1.40]***	1.04 [1.03, 1.05]***
Dual-eligible	1.18 [1.18, 1.19]***	1.20 [1.19, 1.20]***	1.11 [1.10, 1.11]***
**Hospital Characteristics**			
Teaching Hospital			
None	Ref	ref	Ref
Minor teaching hospital	0.27 [0.26, 0.27]***	0.56 [0.56, 0.57]***	0.30 [0.30, 0.30]***
Major teaching hospital	1.09 [1.08, 1.10]***	1.22 [1.21, 1.23]***	1.13 [1.13, 1.14]***
**Community Characteristics**			
Region			
Northeast	Ref	Ref	Ref
Midwest	0.49 [0.49, 0.49]***	0.43 [0.43, 0.43]***	0.71 [0.70, 0.71]***
South	0.38 [0.38, 0.39]***	0.35 [0.34, 0.35]***	0.54 [0.54, 0.55]***
West	0.17 [0. 17, 0. 17]***	0.12 [0.12, 0.12]***	0.45 [0.45, 0.46]***
Urbanicity			
Large central metro	Ref	Ref	Ref
Large fringe metro	1.58 [1.57, 1.59]***	1.14 [1.13, 1.15]***	1.62 [1.61, 1.63]***
Medium/small metro	0.41 [0.41, 0.41]***	0.35 [0.35, 0.36]***	0.77 [0.77, 0.78]***
Micro/non-metropolitan	0.39 [0.38, 0.39]***	0.32 [0.32, 0.33]***	0.75 [0.74, 0.75]***
**Hospital Community Characteristics**			
% Poverty	1.003 [1.001, 1.004]***	1.08 [1.08, 1.08]***	1.00 [1.00, 1.00]
Median household income	0.95 [0.95, 0.95]***	0.95 [0.95, 0.95]***	1.00 [1.00, 1.00]***
Didn’t see doctor because of cost	1.00 [1.00, 1.00]***	1.02 [1.02, 1.02]***	0.99 [0.99, 0.99]***
High housing costs	0.96 [0.96, 0.96]***	1.00 [1.00, 1.00]***	0.94 [0.93, 0.94]***
High school education or higher	0.93 [0.93, 0.94]***	1.01 [1.00, 1.01]***	0.94 [0.94, 0.95]***
Food insecurity	0.98 [0.97, 0.98]***	0.93 [0.93, 0.93]***	1.01 [1.01, 1.01]***
Federally qualified health center/100 K	0.98 [0.98, 0.98]***	0.98 [0.98, 0.98]***	0.99 [0.99, 0.99]***
Primary care / 100 K	1.00 [1.00, 1.00]***	1.00 [1.00, 1.00]***	1.00 [1.00, 1.00]***
Percent population by race			
% White	1.00 [1.00, 1.00]**	1.00 [1.00, 1.01] ***	1.00 [1.00, 1.00]***
% Black	1.01 [1.01, 1.01]***	1.02 [1.01, 1.02]***	1.01 [1.01, 1.01]***
% Hispanic	1.00 [1.00, 1.01]***	1.01 [1.00, 1.01]***	1.01 [1.01, 1.01]***

^***^p < 0.001, **p < 0.01, *p < 0.05

***Notes***. “*ED*” emergency department, “*ACSCs*” ambulatory care sensitive conditions

Results from logistic regression models with robust standard errors, where the outcome is whether the ACSC visit was to a hospital with a high proportion of ACSCs, a high proportion of chronic ACSCs, or a high proportion of acute ACSCs

### Association with safety net status

We ran separate logistic models for each measure of safety net status after adjusting for the patient, hospital, and community characteristics listed in Table 2. Specifically, we examined whether making an ACSC ED visit to a high ACSC hospital was associated with safety net status, with regression results in Table 3. An ACSC ED visit to a hospital in the highest quartile of DSH (versus not) had higher odds of being to a high ACSC hospital and high chronic ACSC hospital (high ACSC hospital aOR: 1.43, 95% CI: 1.42–1.44; high chronic ACSC hospital aOR: 1.59, 95% CI: 1.58–1.60). (Table 3) This relationship was also seen for ACSC ED visits to a high acute ACSC hospital, but to a lesser extent (aOR: 1.02, 1.01–1.03). Similarly, hospitals with the highest proportion of all and chronic ACSCs were substantially more likely to serve the highest proportion of dual-eligible beneficiaries in their EDs. Specifically, an ACSC ED visit to a hospital in the highest quintile for dual-eligible beneficiaries (versus not) had higher odds of being to a high ACSC hospital (aOR: 2.23, 95% CI: 2.21–2.25) and high chronic ACSC hospital (aOR: 2.60, 95% CI:2.58–2.62). While this relationship was also seen in visits to high acute ACSC hospitals, but to a lesser extent (1.48, 95% CI:1.47–1.50) (Table 3). An ACSC ED visit to a for-profit hospital (versus non-profit hospital) had higher odds of being to a high ACSC hospital (aOR: 1.38, 95% CI:1.37–1.39), and visits to a public hospital (versus non-profit hospital had lower odds of being to a high ACSC hospital (aOR: 0.64, 95% CI:0.64–0.65). Similar results were seen for visits to hospitals with a high proportion of chronic ACSC visits (aOR: 1.41, 95% CI: 1.40–1.42 and 0.63, 0.62–0.64, respectively). While directionally similar, the magnitude was lower for visits to hospitals with a high proportion of acute ACSC visits.

**Table 3:** Adjusted Odds of ACSC ED Visits to High ACSC Hospitals, by three definitions of safety net status

	High ACSC Hospital aOR [95% CI]	High Chronic ACSC Hospital aOR [95% CI]	High Acute ACSC Hospital aOR [95% CI]
**DSH Model**			
High DSH	1.43 [1.42, 1.44]***	1.59 [1.58, 1.60]***	1.02 [1.01, 1.03]***
**Dual Eligibility Model**			
High Dual-Eligibility	2.23 [2.21, 2.25]***	2.60 [2.58, 2.62]***	1.48 [1.47, 1.50]***
**Ownership Model**			
Ownership *(Ref Not-for-profit)*			
For-Profit	1.38 [1.37, 1.39]***	1.41 [1.40, 1.42]***	1.17 [1.17, 1.18]***
Public	0.64 [0.64, 0.65]***	0.63 [0.62, 0.64]***	0.94 [0.93, 0.94]***

^***^p < 0.001, **p < 0.01, *p < 0.05

*Notes*. “*ED*” emergency department, “*ACSCs*” ambulatory care sensitive conditions, “*DSH*” disproportionate share

The table shows three different logistic regression models (DSH model, dual eligibility model, and ownership model) examining the association between ACSC ED visits by Medicare patients to hospitals with high proportion of ACSC visits (overall, chronic ACSCs only, or acute ACSCs only), and safety net status. Each of the models has robust standard errors and adjusts for patient, hospital, and hospital community characteristics presented in Table 2, except for individual patient dual-eligibility

The hospital-level correlation between proportion of various ACSC visits, proportion of dual-eligible beneficiaries, and DSH funding varied. (Table 4) The proportion of all ACSC and acute ACSC ED visits (0.76, 95% CI: 0.74–0.77, *p* < 0.001) and all ACSC and chronic ACSC visits (0.88, 95% CI: 0.87–0.89, *p* < 0.001) were strongly correlated, but the proportion of acute and chronic ACSC visits was weak (0.36, 95% CI: 0.33–0.39, *p* < 0.001). There was strong correlation (0.77, 95% CI: 0.76–0.79, *p* < 0.001) between the proportion of dual-eligible beneficiaries and DSH, but weak correlation between the proportion of ACSC ED visits and proportion of dual-eligible beneficiaries and DSH (dual-eligible: 0.29, 95% CI: 0.25–0.32, *p* < 0.001; DSH: 0.2, 95% CI: 0.16–0.24, *p* < 0.001). We observed a statistically insignificant (and negligible) correlation between hospitals in the highest category of DSH and those with the highest proportion of acute ACSC ED visits.

**Table 4:**
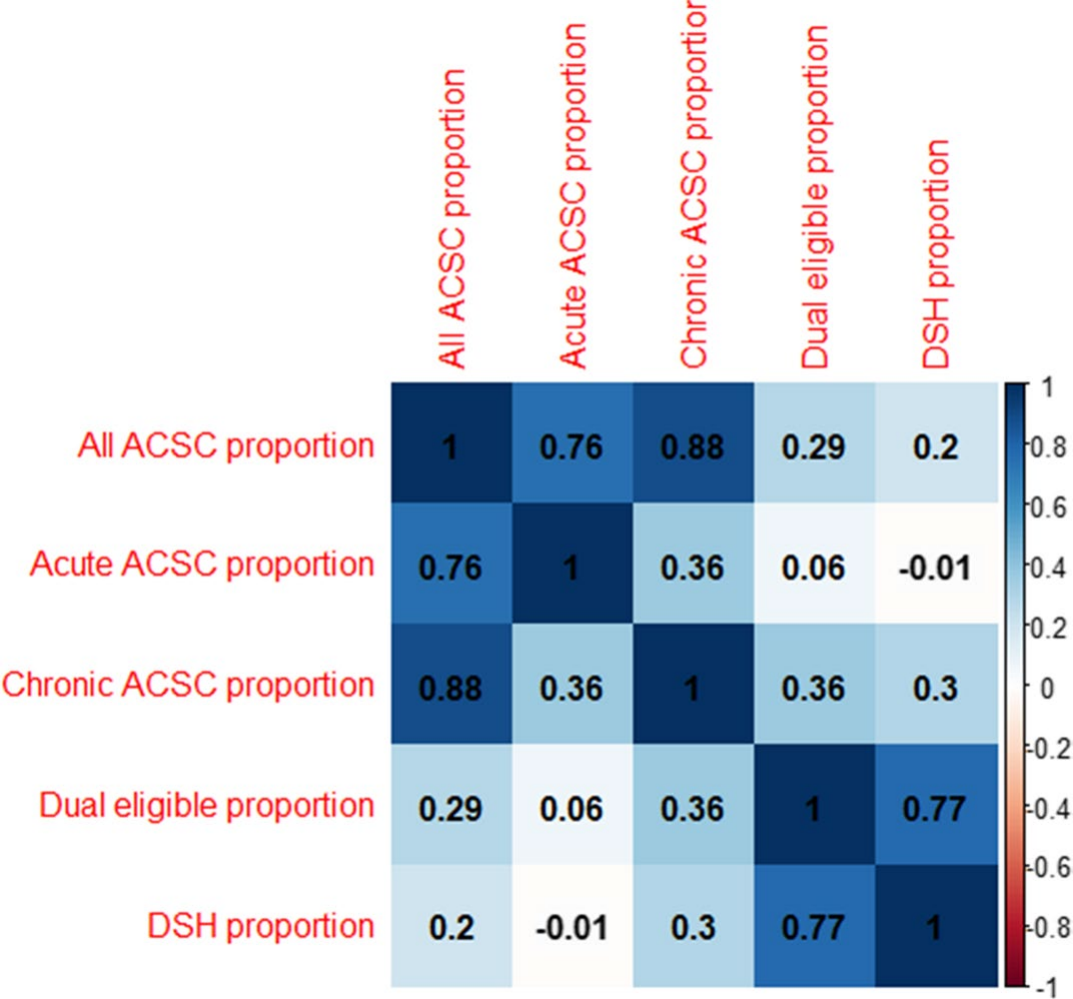
Correlation of Proportions of ACSC ED Visits with DSH Index and Proportion of Dual-Eligible Patients

*Notes*. “*ED*” emergency department, “*ACSCs*” ambulatory care sensitive conditions, “*DSH*” Disproportionate Share Hospital

Correlation matrix based on the proportion at each hospital of all ACSCs, acute ACSCs, chronic ACSCs, the proportion of patients dually-eligible for Medicare and Medicaid, and DSH, each as described in Methods

## Discussion

Previous studies suggested that Medicare patients making ACSC ED visits are more likely to be vulnerable than those who are making non-ACSC ED visits [3, 14]. Our study suggests that even among these patients, those visiting hospitals that see high volumes of ACSCs are more likely to be Black, dually-eligible for Medicare and Medicaid, and have higher comorbidity burden. This suggests that high ACSC hospitals may be serving a particularly disadvantaged and vulnerable patient population. However, our results looking at the association of ACSC visits to high ACSC hospitals and to safety net hospitals and correlation between the proportion of ACSCs and the proportion of dual-eligible patients or DSH proportion suggest a complex relationship between ACSC visits and visits to safety net hospitals.

Specifically, we found that the associations between ACSC visits to high ACSC hospitals and visits to safety net hospitals differed based on the definition of safety net status and the type of ACSC visit. ACSC visits and hospitalizations are commonly seen as potentially preventable ED visits and hospitalizations that reflect a lower quality of outpatient care [1, 4]. The implicit assumption behind inclusion in pay-for-performance programs is that physician groups can reduce ACSC hospitalizations by improving the quality of their outpatient care. Yet the fact that we see a strong association between visits to high ACSC hospitals and visits to safety net hospitals for two definitions, particularly those hospitals treating the highest proportion of dual-eligible patients – even after controlling for patient and community factors – suggests that ED care in hospitals providing the highest proportion of ACSC visits might not be driven by individual gaps in quality of outpatient care that can be modified by outpatient physicians [28].

If ACSC ED visits to high ACSC hospitals reflect poor access to outpatient care, rather than poor quality provided by individual outpatient physicians, it may be important to provide these high ACSC hospitals with extra resources. Care for ACSC visits and hospitalizations are costly for hospitals, over $36 billion in 2021 dollars, [5] and reimbursement rates are lower for ACSC ED visits compared to all ED visits – payments for ACSC ED visits were only 34% of charges, [29] compared to 51% for all ED visits [30]. This means that high ACSC hospitals may be financially burdened for providing ACSC care. It is particularly problematic because hospitals are required to provide treatment for these ACSC ED visits. Specifically, even though ACSCs by definition could have been treated at a lower cost [29] in an ambulatory setting earlier in the disease course, the Emergency Medical Treatment and Labor Act (EMTALA) requires Medicare-participating hospitals to provide emergency care and treatment for these ACSC ED visits. However, our results suggest that the proportion of ACSC visits at a hospital was only weakly correlated with DSH funding, and the proportion of acute ACSC care was negligibly correlated with the DSH index. This is consistent with a previous report by the Medicaid and CHIP Payment and Access Commission report, which found little association between DSH payments received and care of low-income populations, and that up to one-third of hospitals receiving DSH payments did not go to the hospitals with greatest need [31]. Our finding suggests that payment policy may inadequately support these high ACSC hospitals, even though they play a critical role in the safety net.

Our analysis also suggested that other safety net definitions may not adequately capture high ACSC hospitals. For instance, although we observed an association between ACSC visits to high ACSC hospitals and to hospitals with the highest quintile of dual-eligible patients, which previous work suggests may serve patients who are particularly vulnerable, [23] the overall proportion of dual-eligible patients was only weakly correlated with proportion of ACSC visits. Our results also suggest that high ACSC hospitals were less likely to be public hospitals, and more likely to be for-profit hospitals, compared to non-profit hospitals, which may reflect findings from previous studies that geography and market structure may influence service offerings and patient case-mix at hospitals of different ownership [32–35].

Our study also suggested that the type of ACSC (chronic or acute) mattered. These findings suggest that visits to high acute ACSC hospitals in particular had a small association with visits to safety net hospitals.

This study has limitations. First, our results use Medicare fee-for-service data, so may not generalize to non-Medicare fee-for-service populations, including Medicare Advantage. We focused on Medicare because a high percentage of Medicare ED visits are ACSC ED visits [2, 3]. Recent scholarship on ACSC hospitalizations suggest that disparities in ACSC ED visits are particularly pronounced for Black beneficiaries in Medicare Advantage; [36] to our knowledge, studies have not examined whether this relationship is also true for ACSC ED visits. In addition, CMS has a particular interest in ACSCs, [4] and plays a critical role in driving payment policy. We focused on ACSC ED visits as opposed to hospitalizations because hospitals may have different admission thresholds for ACSCs, [37] and we did not want to define a high ACSC hospital based on criteria that could vary by hospital. Thus, our study results may not generalize to ACSC hospitalizations.

Second, the data used in this study are from 2013 and 2014. Despite the relative age of this data, this study is still policy relevant because the geographic distribution of the socioeconomic attributes for these hospitals likely persists over time. Importantly, Medicaid expansion likely has minimal influence on these findings since expansion was to patients under age 65.

Third, this study used several different definitions of safety net hospital to examine the association between visits to high ACSC hospitals and safety net hospitals. However, we are unable to evaluate other safety net hospital definitions such as uncompensated care burden using Medicare claims. Fourth, there may be hospitals that still provide a significant amount of ACSC care that were not captured by our study. For instance, we excluded smaller hospitals because of differences in distribution of ACSC ED visits in these smaller hospitals (Appendix II) and used a 80^th^ percentile threshold to define high ACSC hospitals. However, both smaller hospitals and hospitals providing under the 80^th^ percentile of ACSC visits might still provide a significant amount of ACSC ED care. In sensitivity analyses (Appendix III), we varied the threshold for our definition of high ACSC hospital and obtained similar results, however additional definitions may result in other findings and without clear definitions of safety net status, further explorations may be warranted.

## Conclusion

In conclusion, our study found that EDs that see high volumes of Medicare patients with ACSCs, especially those that see high volumes of patients with chronic ACSC, treat particularly vulnerable patients even among the patients making ACSC ED visits. This suggests that ACSC visits may reflect a population-level gap in outpatient care, and indicates the importance of supporting high ACSC hospitals as they serve a key role in the safety net. However, our results suggest that although high ACSC hospitals may act as safety net hospitals, they were not always classified as safety net hospitals using three different definitions. This was particularly true of high acute ACSC hospitals. High ACSC hospitals act as safety net hospitals; however, existing safety-net funding through DSH may not provide adequate financial support. Future work should more specifically examine ways that these high ACSC hospitals might be better supported.

## Supplementary Information

**Additional file 1: Appendix 1. **Flow Chart of Sample. **Appendix 2. **Distribution of the proportion of ACSC Visits, by Hospital Bed Size. **Appendix 3. **Sensitivity Analyses – Different Cut-Offs.

## Data Availability

The datasets generated and/or analyzed during the current study are not publicly available under data use agreements due to patient confidentiality reasons. Copies of the programs used for the current study are available from Alexis Zebrowski at Alexis.Zebrowski@mountsinai.org upon reasonable request.

## Abbreviations

ACSC: Ambulatory care sensitive condition
aOR: Adjusted odds ratio
CI: Confidence interval
COTH: Council of Teaching Hospitals
DSH: Disproportionate Share Hospital
ED: Emergency department
