# Supplementary material for: Emergency departments in the United States treating high proportions of patients with ambulatory care sensitive conditions: a retrospective cross-sectional analysis
Source: BMC Health Serv Res. 2022 Jul 2;22:854. doi: 10.1186/s12913-022-08240-7 (PMC9250723; doi:10.1186/s12913-022-08240-7)
Supplement: Supplementary file 1 — Additional file 1: Appendix 1. Flow Chart of Sample. Appendix 2. Distribution of the proportion of ACSC Visits, by Hospital Bed Size. Appendix 3. Sensitivity Analyses – Different Cut-Offs. [file 12913_2022_8240_MOESM1_ESM.docx]

**Emergency Departments in the United States Treating High Proportions of Patients with Ambulatory Care Sensitive Conditions: A Retrospective Cross-Sectional Analysis**

Online Appendix

Appendix I. Flow Chart of Sample


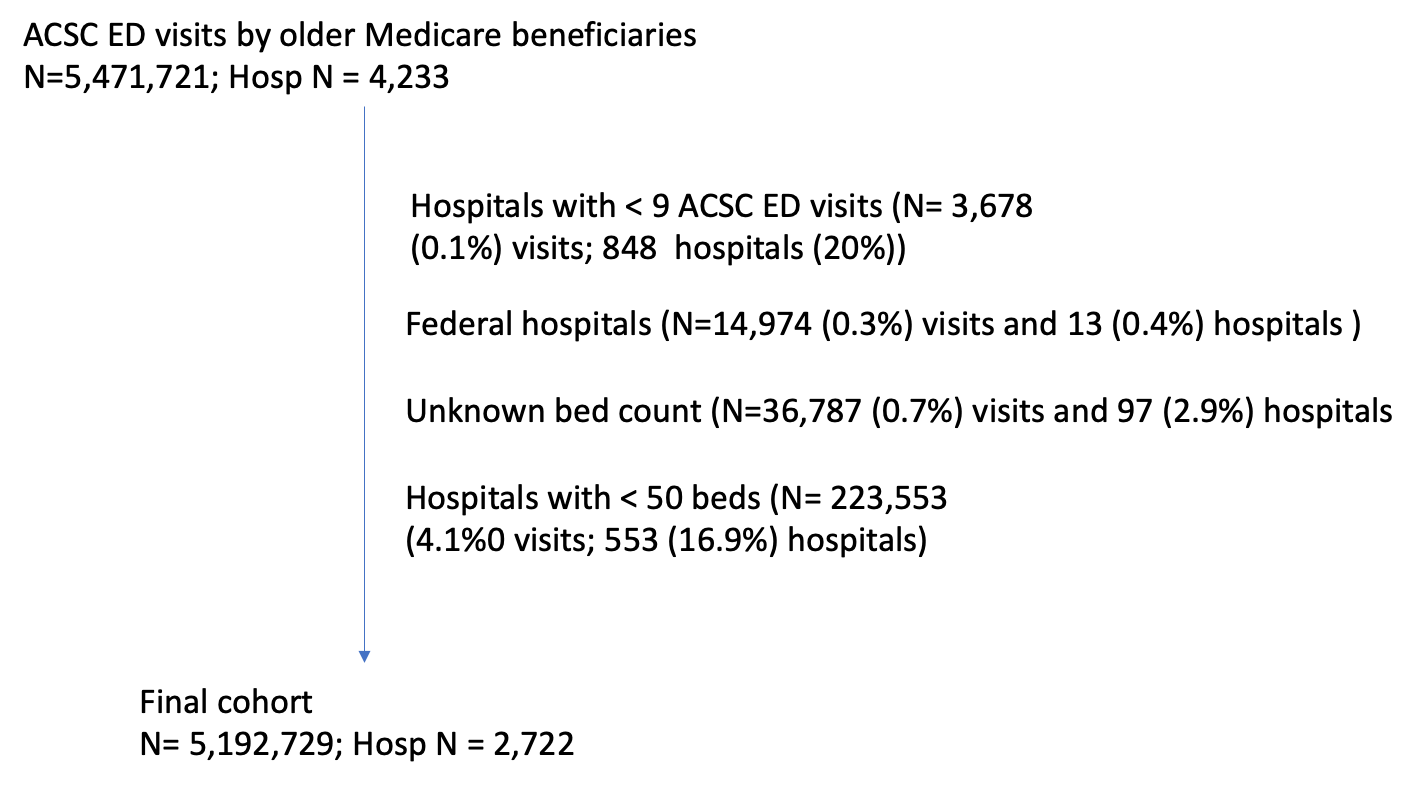


Appendix II

Distribution of the proportion of ACSC Visits, by Hospital Bed Size

eTable 2a. All ACSCs

**
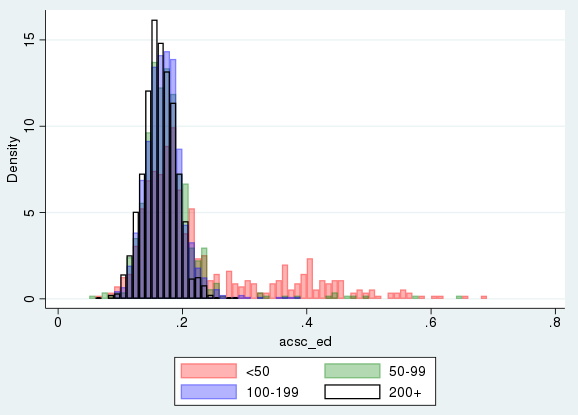
**

eTable 2b. Acute ACSCs

**
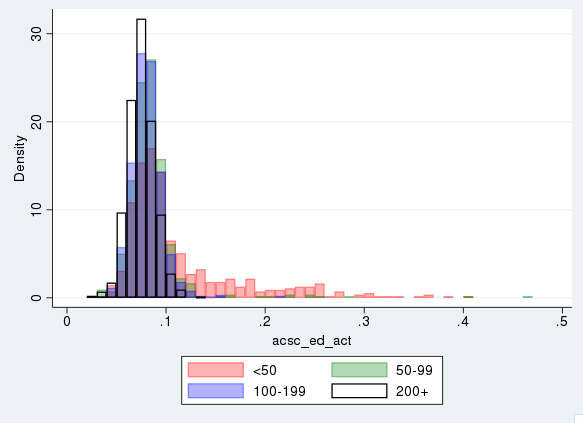
**

eTable 2c. Chronic ACSCs

**
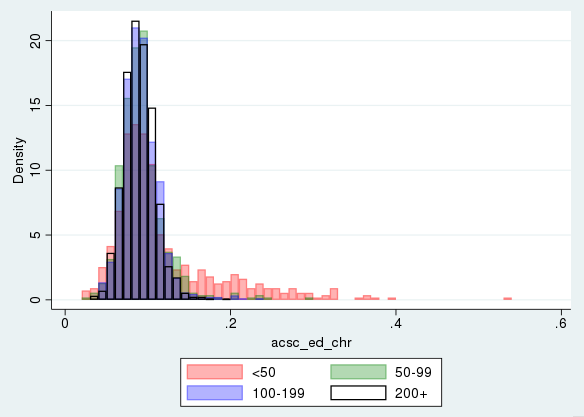
**

**Appendix III**

**Sensitivity Analyses – Different Cut-Offs**

**eTable 1. Sensitivity Analyses with Different Cut-Offs for Defining High ACSC Hospitals -- Patient, Hospital, and Community Factors Associated with an ACSC ED visit to High ACSC Hospitals**

1. **High ACSC Hospital**

|  | 80^th^ Percentile (Main Analysis)  aOR (SE) | 75^th^ Percentile  aOR (SE) | 90^th^ Percentile  aOR (SE) |  |
| --- | --- | --- | --- | --- |
| **Patient Characteristics** |  |  |  |  |
| Age |  |  |  |  |
| 65-74 | Ref | Ref | Ref |  |
| 75-84 | 0.99 (0.003)*** | 0.98 (0.007)** | 0.99 (0.011) |  |
| 85+ | 0.95 (0.003)*** | 0.94 (0.01)*** | 0.98 (0.019) |  |
| Female | 1.02 (0.003)*** | 1.02 (0.007)** | 1.02 (0.010) |  |
| Race |  |  |  |  |
| White | Ref | Ref | Ref |  |
| Black | 1.37 (0.005)*** | 1.27 (0.089)*** | 1.39 (0.147)** |  |
| Other | 1.37 (0.008)*** | 1.26 (0.08)*** | 1.51 (0.137)*** |  |
| Elixhauser comorbidity index |  |  |  |  |
| 0-1 | Ref | Ref | Ref |  |
| 2 | 1.17 (0.005)*** | 1.17 (0.03)*** | 1.12 (0.034)*** |  |
| 3+ | 1.26 (0.004)*** | 1.27 (0.03)*** | 1.23 (0.043)*** |  |
| Dual-eligible | 1.18 (0.003)*** | 1.17 (0.03)*** | 1.22 (0.047)*** |  |
| **Hospital Characteristics** |  |  |  |  |
| Teaching Hospital |  |  |  |  |
| None | Ref | Ref | Ref |  |
| Minor teaching hospital | 0.27 (0.001)*** | 0.36 (0.13)** | 0.18 (0.101)** |  |
| Major teaching hospital | 1.09 (0.003)*** | 1.07 (0.14) | 1.41 (0.267) |  |
| **Community Characteristics** |  |  |  |  |
| Region |  |  |  |  |
| Northeast | Ref | Ref | Ref |  |
| Midwest | 0.49 (0.002)*** | 0.52 (0.096)*** | 0.54 (0.143)* |  |
| South | 0.38 (0.002)*** | 0.42 (0.09)*** | 0.39 (0.097)*** |  |
| West | 0.17 (0.001)*** | 0.19 (0.05)*** | 0.16 (0.062)*** |  |
| Urbanicity |  |  |  |  |
| Large central metro | Ref | Ref | Ref |  |
| Large fringe metro | 1.58 (0.007)*** | 1.50 (0.317) | 1.31 (0.399) |  |
| Medium/small metro | 0.41 (0.002)*** | 0.41 (0.082)*** | 0.33 (0.097)*** |  |
| Micro/non-metropolitan | 0.39 (0.002)*** | 0.42 (0.049)*** | 0.32 (0.0995)*** |  |
| **Hospital Community Characteristics** |  |  |  | |
| % Poverty | 1.003 (0.001)*** | 1.02 (0.03) | 1.03 (0.033) |  |
| Median household income | 0.95 (0.0002)*** | 0.96 (0.011)*** | 0.94 (0.015)*** |  |
| Didn’t see doctor because of cost | 1.00 (0.0005)** | 0.99 (0.022) | 0.97 (0.026)** |  |
| High housing costs | 0.96 (0.0003)*** | 0.97 (0.014)* | 0.96 (0.018)* |  |
| High school education or higher | 0.94 (0.0004)*** | 0.95 (0.024)* | 0.94 (0.022)* |  |
| Food insecurity | 0.98 (0.0009)*** | 0.97 (0.04) | 0.93 (0.048) |  |
| Federally qualified health center/100K | 0.98 (0.0005) | 0.97 (0.020) | 1.00 (0.020) |  |
| Primary care / 100K | 1.00 (0.0001)*** | 1.00 (0.003) | 1.00 (0.004) |  |
| Percent population by race |  |  |  |  |
| % White | 0.999 (0.0002)*** | 1.003 (0.011) | 0.99 (0.014) |  |
| % Black | 1.01 (0.0003)*** | 1.01 (0.012) | 1.00 (0.015) |  |
| % Hispanic | 1.01 (0.0001)*** | 1.01 (0.007) | 1.00 (0.008) |  |

1. **High Chronic ACSC Hospital**

|  | 80^th^ Percentile (Main Analysis)  aOR (SE) | 75^th^ Percentile  aOR (SE) | 90^th^ Percentile  aOR (SE) |  |
| --- | --- | --- | --- | --- |
| **Patient Characteristics** |  |  |  |  |
| Age |  |  |  |  |
| 65-74 | Ref | Ref | Ref |  |
| 75-84 | 0.96 (0.003)*** | 0.96 (0.008)*** | 0.96 (0.012)** |  |
| 85+ | 0.89 (0.003)*** | 0.89 (0.013)*** | 0.90 (0.021)*** |  |
| Female | 0.99 (0.003)** | 1.00 (0.007) | 0.98 (0.011) |  |
| Race |  |  |  |  |
| White | Ref | Ref | Ref |  |
| Black | 1.61 (0.006)*** | 1.55 (0.101)*** | 2.03 (0.19)*** |  |
| Other | 1.28 (0.007)*** | 1.30 (0.082)*** | 1.33 (0.12)** |  |
| Elixhauser comorbidity index |  |  |  |  |
| 0-1 | Ref | Ref | Ref |  |
| 2 | 1.23 (0.005)*** | 1.19 (0.026)*** | 1.14 (0.034)*** |  |
| 3+ | 1.39 (0.005)*** | 1.35 (0.033)*** | 1.27 (0.043)*** |  |
| Dual-eligible | 1.20 (0.003)*** | 1.19 (0.031)*** | 1.28 (0.048)*** |  |
| **Hospital Characteristics** |  |  |  |  |
| Teaching Hospital |  |  |  |  |
| None | ref | ref | Ref |  |
| Minor teaching hospital | 0.56 (0.003)*** | 0.58 (0.158)* | 0.73 (0.229) |  |
| Major teaching hospital | 1.22 (0.004)*** | 1.16 (0.154) | 1.40 (0.277) |  |
| **Community Characteristics** |  |  |  |  |
| Region |  |  |  |  |
| Northeast | Ref | Ref | Ref |  |
| Midwest | 0.43 (0.002)*** | 0.45 (0.083)*** | 0.43 (0.109)*** |  |
| South | 0.35 (0.002)*** | 0.34 (0.067)*** | 0.35 (0.086)*** |  |
| West | 0.12 (0.001)*** | 0.12 (0.031)*** | 0.11 (0.038)*** |  |
| Urbanicity |  |  |  |  |
| Large central metro | Ref | Ref | Ref |  |
| Large fringe metro | 1.14 (0.005)*** | 1.51 (0.32) | 1.18 (0.331) |  |
| Medium/small metro | 0.36 (0.001)*** | 0.38 (0.074)*** | 0.26 (0.076)*** |  |
| Micro/non-metropolitan | 0.32 (0.002)*** | 0.32 (0.076)*** | 0.22 (0.067)*** |  |
| **Hospital Community Characteristics** |  |  |  | |
| % Poverty | 1.08 (0.001)*** | 1.08 (0.028)** | 1.03 (0.034) |  |
| Median household income | 0.95 (0.0002)*** | 0.94 (0.011)*** | 0.93 (0.016)*** |  |
| Didn’t see doctor because of cost | 1.02 (0.001)*** | 1.04 (0.023) | 1.01 (0.028) |  |
| High housing costs | 1.00 (0.0003)*** | 0.99 (0.013) | 0.99 (0.018) |  |
| High school education or higher | 1.01 (0.001)*** | 1.01 (0.024) | 0.95 (0.024) |  |
| Food insecurity | 0.93 (0.0008)*** | 0.89 (0.036)** | 0.91 (0.048) |  |
| Federally qualified health center/100K | 0.98 (0.001)*** | 0.96 (0.023) | 1.02 (0.021) |  |
| Primary care / 100K | 1.00 (0.0001)*** | 1.00 (0.003) | 1.00 (0.004) |  |
| Percent population by race |  |  |  |  |
| % White | 1.01 (0.0002) | 0.99 (0.011) | 0.99 (0.013) |  |
| % Black | 1.001 (0.0002)*** | 1.01 (0.013) | 1.00 (0.015) |  |
| % Hispanic | 1.01 (0.0001)*** | 1.003 (0.007) | 1.00 (0.008) |  |

1. **High Acute ACSC**

|  | 80^th^ Percentile (Main Analysis)  aOR (SE) | 75^th^ Percentile  aOR (SE) | 90^th^ Percentile  aOR (SE) |  |
| --- | --- | --- | --- | --- |
| **Patient Characteristics** |  |  |  |  |
| Age |  |  |  |  |
| 65-74 | Ref | Ref | Ref |  |
| 75-84 | 1.03 (0.003)*** | 1.02 (0.007)** | 1.03 (0.011)** |  |
| 85+ | 1.06 (0.003)*** | 1.06 (0.016)*** | 1.07 (0.020)*** |  |
| Female | 1.04 (0.003)*** | 1.04 (0.007)*** | 1.03 (0.010)*** |  |
| Race |  |  |  |  |
| White | Ref | Ref | Ref |  |
| Black | 0.81 (0.003)*** | 0.78 (0.048)*** | 0.79 (0.075)* |  |
| Other | 1.16 (0.006)*** | 1.11 (0.067) | 1.36 (0.119) *** |  |
| Elixhauser comorbidity index |  |  |  |  |
| 0-1 | Ref | Ref | Ref |  |
| 2 | 1.04 (0.004)*** | 1.04 (0.022) | 1.07 (0.032)* |  |
| 3+ | 1.04 (0.003)*** | 1.02 (0.025) | 1.08 (0.039)* |  |
| Dual-eligible | 1.11 (0.003)*** | 1.08 (0.028)** | 1.14 (0.044)*** |  |
| **Hospital Characteristics** |  |  |  |  |
| Teaching Hospital |  |  |  |  |
| None | Ref | Ref | Ref |  |
| Minor teaching hospital | 0.30 (0.002)*** | 0.34 (0.106)*** | 0.17 (0.093)** |  |
| Major teaching hospital | 1.14 (0.003)*** | 1.17 (0.145) | 1.21 (0.205) |  |
| **Community Characteristics** |  |  |  |  |
| Region |  |  |  |  |
| Northeast | Ref | Ref | Ref |  |
| Midwest | 0.71 (0.003)*** | 0.69 (0.125)* | 0.65 (0.178) |  |
| South | 0.54 (0.002)*** | 0.49 (0.088)*** | 0.45 (0.119) ** |  |
| West | 0.45 (0.002)*** | 0.44 (0.101)*** | 0.63 (0.208) |  |
| Urbanicity |  |  |  |  |
| Large central metro | Ref | Ref | Ref |  |
| Large fringe metro | 1.62 (0.007)*** | 1.25 (0.261) | 1.65 (0.516) |  |
| Medium/small metro | 0.77 (0.003)*** | 0.57 (0.119)** | 0.66 (0.187) |  |
| Micro/non-metropolitan | 0.75 (0.004)*** | 0.59 (0.143)* | 0.61 (0.196) |  |
| **Hospital Community Characteristics** |  |  |  | |
| % Poverty | 1.00 (0.001) | 1.02 (0.028) | 1.05 (0.038) |  |
| Median household income | 1.00 (0.0002)*** | 1.00 (0.010) | 0.99 (0.016) |  |
| Didn’t see doctor because of cost | 0.99 (0.0005)*** | 0.99 (0.020) | 0.96 (0.028) |  |
| High housing costs | 0.94 (0.0002)*** | 0.94 (0.012)*** | 0.94 (0.016)*** |  |
| High school education or higher | 0.95 (0.0004)*** | 0.96 (0.019)* | 0.95 (0.022)* |  |
| Food insecurity | 1.01 (0.0008)*** | 1.03 (0.037) | 1.02 (0.054) |  |
| Federally qualified health center/100K | 0.99 (0.0004)*** | 0.98 (0.017) | 0.99 (0.022) |  |
| Primary care / 100K | 1.00 (0.0001)*** | 1.00 (0.003) | 1.00 (0.003) |  |
| Percent population by race |  |  |  |  |
| % White | 1.01 (0.0002)*** | 1.02 (0.011) | 1.03 (0.020) |  |
| % Black | 1.01 (0.0003)*** | 1.02 (0.012) | 1.04 (0.021) |  |
| % Hispanic | 1.01 (0.0001)*** | 1.01 (0.007) | 1.01 (0.009) |  |

*** p<0.001, **p<0.01, *p<0.05

**Notes**. “ED” = emergency department; “ACSCs” = ambulatory care sensitive conditions

Results from logistic regression models with robust standard errors, where the outcome is whether the ACSC visit was to a hospital with a high proportion of ACSCs, a high proportion of chronic ACSCs, or a high proportion of acute ACSCs. Standard errors are for the aOR.

**eTable 2. Sensitivity Analyses with Different Cut-Offs for Defining High ACSC Hospitals -- Adjusted Odds of ACSC ED Visits to High ACSC Hospitals, by three definitions of safety net status.**

1. **High ACSC Hospital**

|  | 80^th^ Percentile (Main Analysis)  aOR (SE) | 75^th^ Percentile  aOR (SE) | 90^th^ Percentile  aOR (SE) |
| --- | --- | --- | --- |
| **DSH Model** |  |  |  |
| High DSH | 1.43 (0.005)*** | 1.31 (0.213) | 2.14 (0.426)*** |
| **Dual Eligibility Model** |  |  |  |
| High Dual-Eligibility | 2.23 (0.009)*** | 1.61 (0.295)** | 2.36 (0.537)*** |
| **Ownership Model** |  |  |  |
| Ownership  *(Ref Not-for-profit)* |  |  |  |
| For-Profit | 1.38 (0.005)*** | 1.42 (0.227)* | 1.47 (0.306) |
| Public | 0.64 (0.003)*** | 0.71 (0.145) | 0.84 (0.240) |

1. High Chronic ACSC Hospital

|  | 80^th^ Percentile (Main Analysis)  aOR (SE) | 75^th^ Percentile  aOR (SE) | 90^th^ Percentile  aOR (SE) |
| --- | --- | --- | --- |
| **DSH Model** |  |  |  |
| High DSH | 1.59 (0.005)*** | 1.37 (0.217)* | 2.53 (0.480)*** |
| **Dual Eligibility Model** |  |  |  |
| High Dual-Eligibility | 2.60 (0.010)*** | 1.68 (0.300)** | 2.79 (0.602)*** |
| **Ownership Model** |  |  |  |
| Ownership  *(Ref Not-for-profit)* |  |  |  |
| For-Profit | 1.41 (0.005)*** | 1.38 (0.226) | 1.81 (0.387)** |
| Public | 0.63 (0.003)*** | 0.75 (0.158) | - 1. (0.246) |

1. High Acute ACSC Hospital

|  | 80^th^ Percentile (Main Analysis)  aOR (SE) | 75^th^ Percentile  aOR (SE) | 90^th^ Percentile  aOR (SE) |
| --- | --- | --- | --- |
| **DSH Model** |  |  |  |
| High DSH | 1.02 (0.004)*** | 1.05 (0.167) | 1.15 (0.236) |
| **Dual Eligibility Model** |  |  |  |
| High Dual-Eligibility | 1.48 (0.006)*** | 1.15 (0.208) | 1.68 (0.412)* |
| **Ownership Model** |  |  |  |
| Ownership  *(Ref Not-for-profit)* |  |  |  |
| For-Profit | 1.17 (0.004)*** | 1.30 (0.188) | 1.00 (0.209) |
| Public | 0.04 (0.004)*** | 0.93 (0.174) | 0.72 (0.190) |

*** p<0.001, **p<0.01, *p<0.05

**NOTES**. “ED” = emergency department; “ACSCs” = ambulatory care sensitive conditions; “DSH” = disproportionate share

The tables shows three different logistic regression models (DSH model, dual eligibility model, and ownership model) examining the association between ACSC ED visits by Medicare patients to hospitals with high proportion of ACSC visits (overall, chronic ACSCs only, or acute ACSCs only), and safety net status. Each of the models has robust standard errors and adjusts for patient, hospital, and hospital community characteristics presented in Table 2, except for individual patient dual-eligibility. Standard errors are for the aOR.
